# Virus-encoded miRNAs in Ebola virus disease

**DOI:** 10.1038/s41598-018-23916-z

**Published:** 2018-04-24

**Authors:** Janice Duy, Anna N. Honko, Louis A. Altamura, Sandra L. Bixler, Suzanne Wollen-Roberts, Nadia Wauquier, Aileen O’Hearn, Eric M. Mucker, Joshua C. Johnson, Joshua D. Shamblin, Justine Zelko, Miriam A. Botto, James Bangura, Moinya Coomber, M. Louise Pitt, Jean-Paul Gonzalez, Randal J. Schoepp, Arthur J. Goff, Timothy D. Minogue

**Affiliations:** 1 Diagnostic Systems Division, U.S. Army Medical Research Institute of Infectious Diseases, Fort Detrick, Frederick, MD USA; 2 Virology Division, U.S. Army Medical Institute of Infectious Diseases, Fort Detrick, Frederick, MD USA; 3 Metabiota, Kenema, Sierra Leone; 4 Metabiota, Washington, DC USA; 5 Present Address: MRIGlobal – Global Health Surveillance and Diagnostics, Gaithersburg, MD USA; 6 Present Address: Center of Excellence for Emerging & Zoonotic Animal Disease, Kansas State University, Manhattan, KS USA

## Abstract

Ebola virus (EBOV) is a negative-strand RNA virus that replicates in the cytoplasm and causes an often-fatal hemorrhagic fever. EBOV, like other viruses, can reportedly encode its own microRNAs (miRNAs) to subvert host immune defenses. miRNAs are short noncoding RNAs that can regulate gene expression by hybridizing to multiple mRNAs, and viral miRNAs can enhance viral replication and infectivity by regulating host or viral genes. To date, only one EBOV miRNA has been examined in human infection. Here, we assayed mouse, rhesus macaque, cynomolgus macaque, and human samples infected with three EBOV variants for twelve computationally predicted viral miRNAs using RT-qPCR. Ten miRNAs aligned to EBOV variants and were detectable in the four species during disease with several viral miRNAs showing presymptomatic amplification in animal models. miRNA abundances in both the mouse and nonhuman primate models mirrored the human cohort, with miR-1-5p, miR-1-3p, and miR-T3-3p consistently at the highest levels. These striking similarities in the most abundant miRNAs during infection with different EBOV variants and hosts indicate that these miRNAs are potential valuable diagnostic markers and key effectors of EBOV pathogenesis.

## Introduction

The most recent outbreak of Ebola virus disease (EVD) in West Africa in 2013–2016 caused over 28,000 reported cases and greater than 11,000 deaths^1^, highlighting the need for accurate and rapid diagnostic tests for patient triage and management of disease spread^2^. The gold standard for diagnosis of EVD is the amplification of viral sequences using RT-PCR. Several assays that target different Ebola virus (EBOV) genes are now available^3^, but the detection of virus in blood samples typically occurs after the onset of clinical symptoms^4,5^. A definitive diagnosis prior to this time can only be achieved by a more sensitive assay, or by targeting other indicators of disease that are more abundant at earlier time points. As an example of the latter, we recently reported a proof-of-concept classifier of EVD using eight host-derived microRNAs (miRNAs) that could potentially be used for presymptomatic diagnosis^6^.

miRNAs are short ~22-nt noncoding RNA species that can post-transcriptionally regulate multiple mRNAs, typically by binding a stretch of 6–8 nts at the 3′ untranslated region. miRNA biogenesis traditionally begins in the nucleus, where a long RNA sequence containing the miRNA in a hairpin (primary miRNA, pri-miRNA) is transcribed and recognized by the complex of Drosha (an RNase III enzyme) and DGCR8 (a dsRNA-binding protein). This is cleaved into a shorter ~70-nt stem-loop structure called the precursor miRNA (pre-miRNA) and translocated by Exportin-5 into the cytoplasm, where it is processed by the endonuclease Dicer into the mature miRNA form. Only recently has it been proven that RNA viruses that do not have a nuclear DNA stage in their replication cycle (such as EBOV) can indeed encode their own miRNAs without compromising viral replication^7–10^.

To date, several groups have predicted that EBOV encodes its own miRNAs, which may be detectable in circulation prior to the viral genome. Liang *et al*.^11^ first reported on potential EBOV-encoded miRNAs by predicting two pre-miRNAs and three mature miRNAs in two species, Sudan virus (SUDV) and EBOV. *In silico* analysis showed that these pre-miRNAs were conserved in two SUDV variants and in six EBOV variants. Further, they confirmed mature miRNA production in cell culture by transfecting the two pre-miRNA sequences, and determined that the production of the mature miRNAs was significantly reduced in Dicer-deficient cells. These results suggest that EBOV miRNA generation is dependent on cellular miRNA processing machinery. The three mature miRNAs were also able to effectively silence their target mRNA sequences in cell culture.

Other groups demonstrated similar findings, with a second report using 102 EBOV/Makona genome sequences from the 2014 outbreak to predict four pre-miRNAs and seven mature miRNA candidates^12^. This group computationally predicted 138 gene targets and their related signaling pathways for these miRNAs. They also showed that synthetic EBOV miRNAs can regulate the expression of several predicted target genes in HeLa cells. A third group used the EBOV/Boende-Lokolia variant^13^ from the 2014 outbreak to predict one pre-miRNA encoding for two mature miRNAs^14^. They confirmed that these miRNAs were produced in mammalian cell lines by transfecting the pre-miRNA sequence into these cells, and further demonstrated *in vitro* that one of the miRNAs (miR-1-5p) suppresses the expression of importin-α5, a nuclear transport protein that interacts with EBOV and may influence viral virulence *in vivo*.

Recently, Chen *et al*.^15^ predicted three pre-miRNAs using the EBOV/Yambuku-Mayinga sequence and retained one of these after alignment with 125 EBOV genomes from the 2014 viral outbreak. This pre-miRNA produces one mature miRNA sequence, miR-VP-3p, which was detected in the serum of EBOV-positive patients during the acute infection phase, but not during the convalescence phase (virus-negative by PCR). Further, miR-VP-3p was detectable in the serum of six virus-negative patients who were admitted for EVD-like symptoms and eventually tested positive for EBOV 1–2 days after initial evaluation. Since miR-VP-3p was found mostly in the exosomal fraction of patient serum, the authors postulated that it was released into circulation prior to the viral genome.

In this work, we investigated whether these putative miRNAs are detectable during EBOV infection using RT-qPCR. We targeted 12 candidate miRNAs with locked nucleic acid (LNA)-based primer sets, and tested for these miRNAs in EBOV cell culture extracts. Examination of nonhuman primate (NHP) plasma/serum samples from two separate EBOV infection cohorts allowed for selection of the most diagnostically relevant viral miRNAs. We also tested whole blood samples collected daily from a BALB/c mouse model of EVD to determine if virus-encoded miRNAs were detectable in this species, and if these miRNAs could be amplified presymptomatically. Finally, we analyzed 15 human samples from the 2014 EBOV/Makona outbreak in Sierra Leone to verify translatability of our findings to a human cohort.

## Results

### Candidate miRNA sequence alignments to EBOV genomes

We performed a BLAT search in the UCSC Ebola Genome Browser (with default reference sequence Ebola virus/H.sapiens-wt/SLE/2014/Makona-G3686.1) for putative EBOV pre-miRNA and mature miRNA sequences as four independent groups have predicted 12 viral miRNAs from different reference genomes: Liang *et al*.^11^ used SUDV and EBOV/Yambuku-Mayinga, Teng *et al*.^12^ used EBOV/Makona, Chen *et al*.^15^ used EBOV/Yambuku-Mayinga, and Liu *et al*.^14^ used EBOV/Boende-Lokolia. The Genome Browser uses the EBOV/Makona KM034562.1 reference sequence, and aligns 158 Ebola genomes from outbreaks spanning 1976–2014, as well as two *Marburgvirus* genomes for comparison.

**Figure 1:**
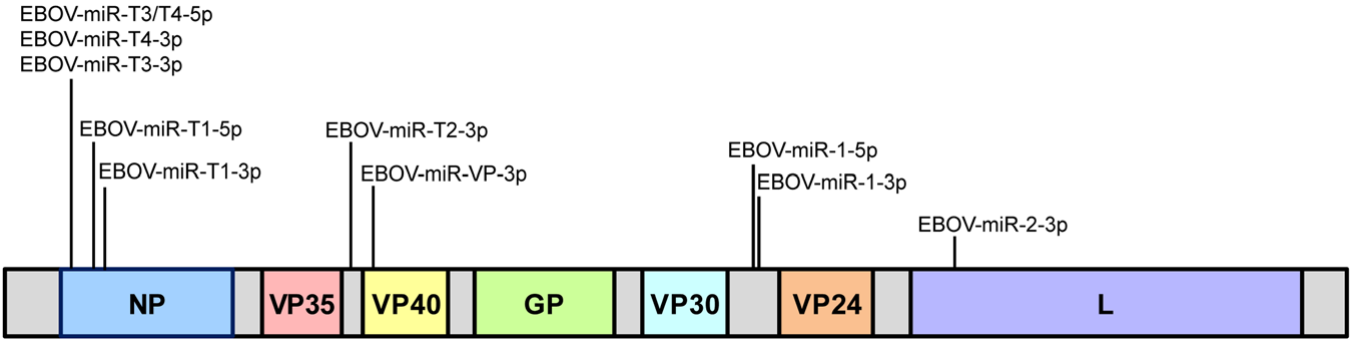
Ten of twelve candidate EBOV miRNAs align to viral genome. Putative miRNA sequences were aligned to the UCSC Ebola Genome Browser using a BLAT search of the *Zaire ebolavirus* isolate Ebola virus/H.sapiens-wt/SLE/2014/Makona-G3686.1 reference sequence, with relative positions denoted. Genome segments are not drawn to scale.

The BLAT search revealed significant alignments to putative EBOV pre-miRNAs and mature miRNAs (Fig. 1). Candidate EBOV pre-miRNA and mature miRNA sequences and their locations in the consensus genome are given in Supplementary Table S1. All miRNAs except miR-VP-3p derived from the complementary RNA (cRNA) strand, which is the coding (mRNA) strand. Examination of the genome alignment revealed a single G to A substitution in the EBOV-miR-T4-3p sequence at position 572 of the reference, which is the 19^th^ nucleotide of the miRNA sequence.

A BLAT search of EBOV-miR-1-3p (Liang) and EBOV-miR-1-5p (Liang) did not produce a significant alignment, and NCBI BLAST results showed that these sequences were only found in SUDV. Similarly, miR-1-5p was not initially found in the UCSC reference but closer inspection of the pre-miR-1 annotation revealed that miR-1-5p spanned genome position 9892–9913, with a G to A substitution at position 9911 of the EBOV/Makona variant (20^th^ nucleotide of the miRNA sequence); no mismatches were present in the genomes of other EBOV variants.

Further inspection using the Genome Browser revealed the same sequence alignments in the mouse-adapted variant EBOV/May-MA genome, except for a single A to G mismatch at position 683 for miR-T1–5p; this corresponds to the fifth nucleotide in the sequence and is part of the miRNA seed.

### Primer set testing and downselection

**Figure 2:**
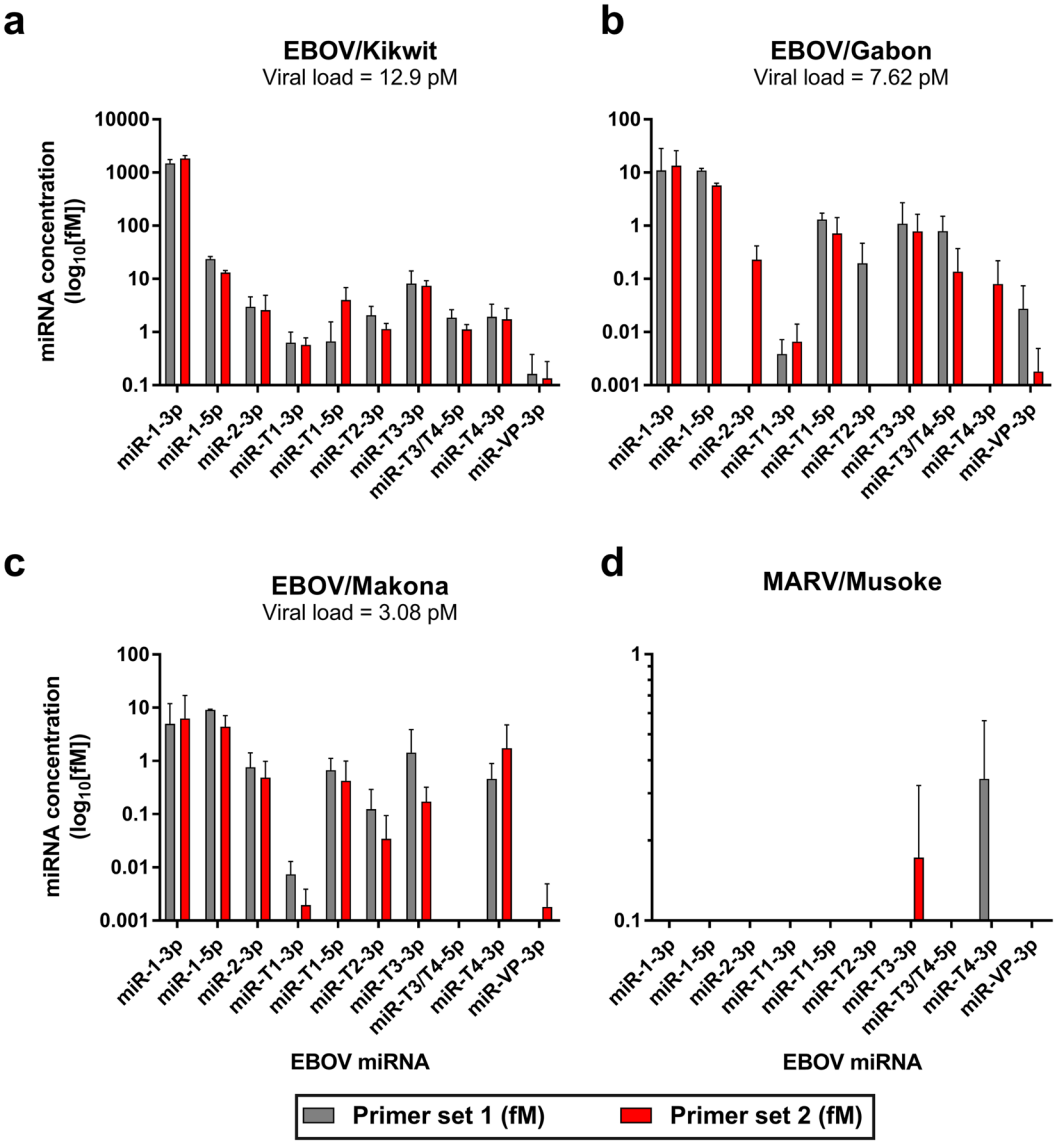
EBOV-encoded miRNAs are present in cell culture supernatants from different EBOV variants. Cell culture supernatant RNA from 10^6^ PFU/mL of (**a**) EBOV/Kikwit, (**b**) EBOV/Gabon, (**c**) EBOV/Makona, and (**d**) MARV/Musoke viral stocks were tested for candidate EBOV miRNAs. Data are presented as the mean ± SD of technical triplicates. EBOV viral concentration was determined by RT-qPCR of a glycoprotein sequence and miRNAs were assayed using custom LNA-based primer sets from Exiqon, Inc. following manufacturer’s protocols. Each putative miRNA was tested with the top two primer sets from the manufacturer’s web design tool.

We selected LNA-based miRNA primer sets because of demonstrated superior selectivity and sensitivity in multiple studies^16–18^. The top two LNA-based primer sets designed by the Exiqon web tool were tested with corresponding synthetic miRNAs and a water-only cDNA control (non-template control, NTC) to assess performance and nonspecific binding. All primers detected the intended targets with low variability and only primer miR-T4-3p-1 showed nonspecific amplification (Cq < 40 for NTC). Therefore, we tested all primer sets with EBOV/Kikwit cell culture supernatant RNA. At 10^6^ PFU/mL of virus (highest concentration tested), 20 of the 24 primer sets showed detection for ten of the twelve reported EBOV miRNAs (Fig. 2). As expected, primers designed to miR-1-3p (Liang) and miR-1-5p (Liang), which only aligned to SUDV variants, showed no target amplification (data not shown).

The abundances of different EBOV miRNAs varied greatly within the same sample. In viral cell culture supernatants, average viral loads for 10^6^ PFU/mL each of EBOV/Kikwit, EBOV/Gabon, and EBOV/Makona were 12.9 pM, 7.62 pM, and 3.08 pM, respectively. For all primer sets tested, viral miRNA amounts were highest in the EBOV/Kikwit sample, trailed by EBOV/Gabon, then EBOV/Makona (Fig. 2, note scale). miR-1-3p, miR-1-5p, and miR-T3-3p were the most abundant in all variants tested. All miRNAs amplified in EBOV/Kikwit, while in EBOV/Gabon two primer sets (miR-2-3p-1 and miR-T2-3p-2) did not amplify their targets and miR-T4-3p-1 showed Cq values similar to those from the NTC. In EBOV/Makona, both primer sets targeting miR-T3/T4-5p, as well as miR-VP-3p-1, showed no amplification. Primer miR-T4-3p-2 showed late-cycle amplification (Cq = 35.08) in one out of three replicates, with no detection in the remaining two replicates. We included cell culture supernatants from the related MARV/Musoke for preliminary cross-reactivity testing. No primer sets showed amplification except for miR-T3-3p-2 and miR-T4-3p-1, which also displayed amplification in the water-only control samples.

**Figure 3:**
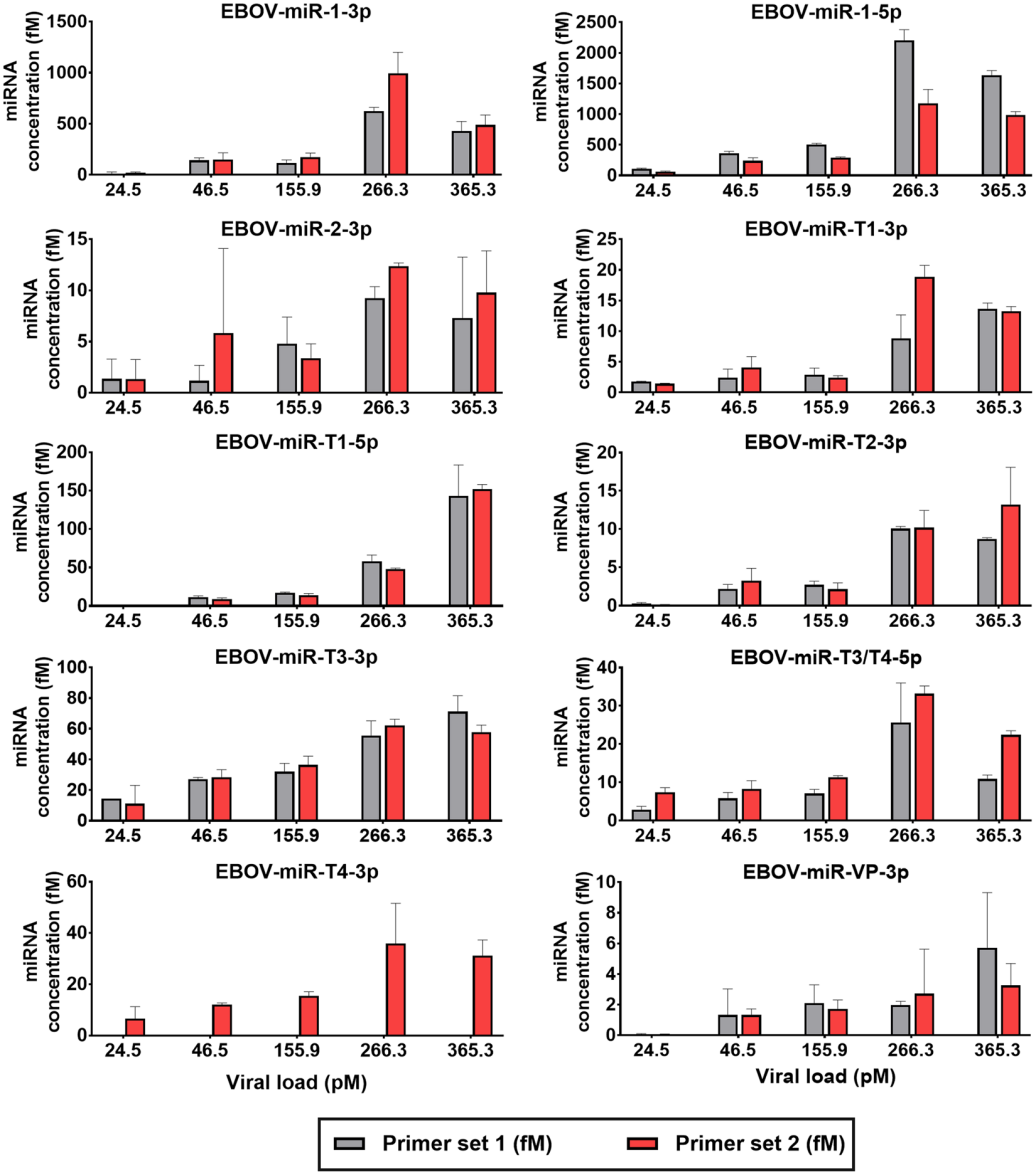
EBOV-encoded miRNAs were detectable in the plasma of EBOV/Kikwit-infected NHPs. Ten candidate miRNAs were detected by 19 LNA primer sets in rhesus macaque plasma from an EBOV/Kikwit infection cohort (NHP n = 3). Data are presented as the mean ± SD of biological replicates run in technical triplicate. miRNAs were assayed with custom LNA primer sets obtained from Exiqon, Inc. following manufacturer’s instructions (see Methods for details). Each miRNA target was amplified by two separate primer sets. Viral load was determined using an RT-qPCR assay which amplifies part of the viral glycoprotein sequence.

In subsequent analysis, we tested all primer sets with plasma samples with known EBOV titers from three rhesus macaques infected with EBOV/Kikwit through aerosol exposure (sample n = 14, Fig. 3). All putative miRNAs were detected in these samples, with miR-1-5p and miR-1-3p being most abundant, followed by miR-T3-3p, miR-T1-5p, miR-T4-3p, and miR-T3/T4-3p. While still detectable, miR-T1-3p, miR-2-3p, miR-T2-3p, and miR-VP-3p presented at far lower concentrations. Importantly, no primers amplified miRNAs in known virus-negative plasma samples (day -7 and day 0 relative to EBOV exposure) with the exception of miR-T4-3p-1.

While the most diagnostically relevant (most abundant) miRNAs are miR-1-3p, miR-1-5p, and miR-T3-3p based on these results, we carried the best-performing primer sets into further analysis for each miRNA to account for the diversity of our sample sets, which encompasses different species and EBOV variants.

### EBOV miRNAs in longitudinally-collected NHP samples

To determine if EBOV miRNAs can be used to diagnose EVD in the NHP model, we tested longitudinally collected, archived plasma samples from two separate studies. The first set was from six rhesus macaques exposed to EBOV/Kikwit, including samples from the three NHPs tested for primer set downselection. The second sample set included three cynomolgus macaques infected with EBOV/Makona.

All EBOV miRNAs were detectable in EBOV/Kikwit rhesus macaque samples at symptomatic timepoints (Table 1 and Supplementary Fig. S1a). In this cohort, miR-1-5p and miR-1-3p yielded the highest concentrations, followed by miR-T1-5p and miR-T3-3p. Viral load for this infection model increased from day 6 to day 7 or from day 6 to day 8 with all miRNA concentrations rising concomitantly.

**Table 1:** EBOV miRNAs were detected in rhesus macaques exposed to aerosolized EBOV/Kikwit.

EBOV miRNA	miRNA concentration by day post-exposure (fM)					
	Day −7 (EBOV load ND)	Day 0 (ND)	Day 3 (ND)	Day 6 (51.71 pM)	Day 7 (287.17 pM)	Day 8 (295.55 pM)
miR-1-3p	ND	ND	ND	58.37 ± 20.57	557.04 ± 111.82	245.68 ± 48.88
miR-1-5p	ND	ND	ND	216.36 ± 15.75	1424.69 ± 108.41	1151.34 ± 72.38
miR-2-3p	ND	ND	ND	1.74 ± 1.32	8.02 ± 1.68	5.20 ± 3.76
miR-T1-3p	ND	ND	ND	2.13 ± 0.52	12.04 ± 1.11	9.94 ± 0.55
miR-T1-5p	ND	ND	ND	4.64 ± 1.06	31.27 ± 1.44	87.08 ± 5.19
miR-T2-3p	ND	ND	ND	0.98 ± 0.36	5.95 ± 0.23	5.51 ± 0.64
miR-T3-3p	ND	ND	ND	17.85 ± 3.27	38.15 ± 8.54	52.42 ± 8.10
miR-T3/T4-5p	ND	ND	ND	7.02 ± 1.39	22.00 ± 2.37	17.83 ± 1.39
miR-T4-3p	ND	ND	ND	8.07 ± 2.23	22.49 ± 8.60	22.56 ± 4.73
miR-VP-3p	ND	ND	ND	0.70 ± 0.62	1.05 ± 0.20	2.93 ± 1.90

**Note:** Data are presented as the mean ± SD of biological replicates assayed in technical triplicate. The corresponding EBOV load at each timepoint is given in parentheses below each day. **ND** = not detected.

Similarly, miR-1-5p was most abundant in the EBOV/Makona group, followed by miR-T3-3p, miR-T4-3p, miR-T3/T4-5p, and miR-1-3p (Table 2 and Supplementary Fig. S1b). Circulating virus peaked and then declined after day 6 with viral miRNAs following two trends: (1) mirroring viral load (miR-1-3p, miR-1-5p, miR-2-3p, miR-T1-5p) and (2) increasing over time independent of viral load (miR-T1-3p, miR-T2-3p, miR-T3-3p, miR-T3/T4-5p, miR-T4-3p, miR-VP-3p). In contrast to the EBOV/Kikwit cohort, one technical replicate in one NHP showed presymptomatic amplification of the most-abundant miR-1-5p at day 3 without direct detection of EBOV (miRNA Cq = 34.79, NTC Cq not detected after 45 cycles). In both rhesus and cynomolgus macaque models, miRNAs at the lowest concentrations were miR-2-3p, miR-T2-3p, and miR-VP-3p.

**Table 2:** EBOV miRNAs were detected in cynomolgus macaques intramuscularly injected with EBOV/Makona.

EBOV miRNA	miRNA concentration by day post-exposure (fM)				
	Day −1 (EBOV load ND)	Day 3 (ND)	Day 6 (10.01 pM)	Day 9 (1.35 pM)	Day 10 (4.19 pM)
miR-1-3p	ND	ND	9.95 ± 15.74	2.74 ± 2.79	35.69 ± 31.42
miR-1-5p	ND	0.37 ± 0.64	250.82 ± 22.56	203.89 ± 15.91	328.52 ± 34.95
miR-2-3p	ND	ND	2.11 ± 1.40	1.40 ± 0.913	6.96 ± 7.69
miR-T1-3p	ND	ND	1.60 ± 0.39	4.47 ± 0.50	5.81 ± 0.85
miR-T1-5p	ND	ND	4.20 ± 1.23	1.96 ± 0.72	4.91 ± 1.52
miR-T2-3p	ND	ND	0.68 ± 0.30	1.17 ± 1.23	2.39 ± 0.92
miR-T3-3p	ND	ND	19.62 ± 10.17	50.21 ± 5.74	59.64 ± 10.12
miR-T3/T4-5p	ND	ND	10.56 ± 2.24	30.60 ± 7.27	36.44 ± 2.32
miR-T4-3p	ND	ND	12.06 ± 4.48	29.20 ± 5.80	38.83 ± 6.22
miR-VP-3p	ND	ND	0.02 ± 0.02	0.23 ± 0.40	0.24 ± 0.27

**Note:** Data are presented as the mean ± SD of biological replicates assayed in technical triplicate. Values in bold font indicate amplification in only one of three biological samples, with mean ± SD only for that sample. The corresponding EBOV load at each timepoint is given in parentheses below each day. **ND** = not detected.

Average peak circulating virus levels were 10.7 times higher in the EBOV/Kikwit samples compared to those from EBOV/Makona (peak titers ranged from 12.7 to 365 pM and 2.1 to 32 pM, respectively). Corresponding mean miRNA abundances in EBOV/Kikwit were 5.2 times higher than those from EBOV/Makona. The concentration of the most-abundant miR-1-5p was only 54-fold lower than circulating virus in the EBOV/Makona cohort despite the G-to-A substitution in the 20^th^ nucleotide of the EBOV/Makona sequence; it was ~257-fold lower than virus in the EBOV/Kikwit group. We tested the miR-1-5p-1 primer set with synthetic mismatched RNA to assess the effect of the non-seed mutation and found no differences in amplification efficiency compared to the perfect-match sequence, indicating both variants are equivalently detected (data not shown).

### EBOV miRNAs in mouse whole blood collected at early infection timepoints

We next assayed mouse whole blood samples collected during daily terminal bleeds for all EBOV miRNAs to determine whether these miRNAs are: (1) detectable during infection in a different mammalian model and (2) viable presymptomatic markers of disease. We found that viral miRNAs were also present in the EBOV/May-MA mouse model of infection (Table 3 and Supplementary Fig. S2). Nonspecific amplification at late cycles (Cq > 37) was observed in known virus-free (day 0) samples and was used as the new baseline for this set (data not shown).

**Table 3:** EBOV miRNAs were detected in BALB/c mice intraperitoneally injected with mouse-adapted EBOV/Mayinga.

EBOV miRNA	miRNA concentration by day post-exposure (fM)					
	Day 0 (EBOV load ND)	Day 1 (4.17E-05 pM)	Day 2 (0.04 pM)	Day 3 (3.99 pM)	Day 4 (20.20 pM)	Day 7 (0.22 pM)
miR-1-3p	ND	ND	ND	8.82 ± 6.92	20.94 ± 14.68	51.32 ± 37.27
miR-1-5p	ND	ND	1.08 ± 0.99	8.52 ± 1.51	25.11 ± 2.55	129.37 ± 6.68
miR-2-3p	ND	ND	ND	2.40 ± 1.23	5.00 ± 1.11	12.41 ± 2.36
miR-T1-3p	ND	ND	ND	0.74 ± 0.16	1.78 ± 0.41	18.44 ± 1.83
miR-T1-5p	ND	ND	ND	ND	14.57 ± 8.14*	ND
miR-T2-3p	ND	ND	ND	ND	2.49 ± 0.77	1.26 ± 0.92
miR-T3-3p	ND	ND	0.83 ± 0.82	3.45 ± 2.86	12.46 ± 6.77	101.75 ± 10.34
miR-T3/T4-5p	ND	ND	0.07 ± 0.12	2.32 ± 0.76	3.74 ± 0.92	46.81 ± 3.31
miR-T4-3p	ND	ND	1.19 ± 2.06	3.67 ± 1.16	6.25 ± 3.87	55.58 ± 7.26
miR-VP-3p	ND	ND	ND	0.05 ± 0.10	0.30 ± 0.46	0.62 ± 1.07

**Note:** Data are presented as the mean ± SD of biological replicates assayed in technical triplicate. Values in bold font indicate amplification in only one of three biological samples, with mean ± SD only for that sample. The corresponding EBOV load at each timepoint is given in parentheses below each day. **ND** = not detected. *Value given is based on the mismatched miR-T1-5p sequence.

In this cohort, the EBOV titer remained low until day 3, peaked at day 4, and declined by day 7 post-exposure, with levels lower than those seen during NHP infection (4.17E-05 to 36 pM). Similar to the NHP findings, the miRNAs at the highest levels were miR-1-5p and miR-T3-3p, followed by miR-1-3p, miR-T4-3p, and miR-T3/T4-5p. Inspection of the data revealed miRNA expression trends that could be grouped into: (1) miRNAs that increased steadily during disease (miR-1-3p, miR-2-3p, and miR-VP-3p); (2) miRNAs that remained low until day 4, then increased on day 7 (miR-1-5p, miR-T1-3p, miR-T3-3p, miR-T3/T4-5p, and miR-T4-3p); and (3) one miRNA that was highest on day 4/peak viremia (miR-T2-3p). Analogous to the NHP cohorts, the least abundant miRNAs were miR-2-3p, miR-T2-3p, and miR-VP-3p. Mouse samples from day 0 onward showed low levels of amplification for miR-T1-5p (Cq values ranging from 34.94 to 40) despite the A-to-G mismatch in the seed sequence of the mouse-adapted viral variant. We tested the miR-T1-5p-2 primer set with the synthetic mismatched miRNA and found that the primer set amplified the mouse-adapted sequence, albeit at an average of a 60-fold reduction in efficiency (data not shown). Examination of the melt curves showed different characteristics for the mouse-adapted amplicon, which suggests either abolishment of miR-T1-5p or low levels of nonspecific amplification for this assay. Analysis of the effect of the A-to-G substitution in the seed sequence revealed significant differences between functionalities of the two sequences, with a biological process similarity score of 0.234 (a score of 1 represents identical functionality) calculated using miRmut2GO^19^.

In the mouse samples, average viral load was 5.22 ± 0.27 pM, with miR-1-5p concentrations ~400-fold lower over the course of infection. The EBOV glycoprotein sequence amplified as early as day 1 post-exposure, and was detectable before any miRNAs. However, miR-1-5p, miR-T3-3p, miR-T3/T4-5p, and miR-T4-3p were detectable in three different mice as early as day 2 post-exposure, when animals were asymptomatic for EVD. miR-T3-3p and miR-T3/T4-5p amplified in the same mouse on this day. In contrast to the other miRNAs which were amplified starting on day 3, miR-T2-3p (second-least abundant) was detectable only on days 4 and 7 post-infection.

### EBOV miRNAs in human samples from the 2014 outbreak

To assess the applicability of targeting EBOV-encoded miRNAs in clinical samples, we tested human plasma and serum samples acquired in Sierra Leone in 2014 (Table 4 and Supplementary Fig. S3). Samples were collected 2–27 days post-symptom onset (self-reported by patients during admission). All samples were PCR-positive for EBOV, with titers ranging from 2.1 × 10^−5^ pM to 7.64 pM (equivalent to 19.4 PFU/mL to 6.58 × 10^5^ PFU/mL, data not shown), with an average of 1.06 ± 2.65 pM.

**Table 4:** EBOV miRNAs were present in human samples during infection with EBOV/Makona.

EBOV miRNA	miRNA concentration by patient sample designator (fM)								
	G1 (0.031 pM)	G2 (0.367 pM)	G3 (0.336 pM)	G4 (0.012 pM)	G5 (7.64 pM)	G6 (7.50 pM)	G8 (3.47E-05 pM)	G9 (1.61E-04 pM)	G14 (1.04E-04 pM)
miR-1-3p		**2.67** ± 4.62*		*21.74* ± *23.92*	15.87 ± 10.68	21.78 ± 10.63			
miR-1-5p	0.48 ± 0.11	110.09 ± 1.86	4.17 ± 0.87		361.67 ± 48.69	217.95 ± 5.70	*1.39* ± *0.95*	*0.21* ± *0.26*	
miR-2-3p		0.86 ± 0.74			1.38 ± 0.58	1.84 ± 0.25			
miR-T1-3p		4.62 ± 1.02	0.38 ± 0.17	1.36 ± 0.45	9.42 ± 1.41	5.03 ± 2.31			
miR-T1-5p		0.58 ± 0.99			4.55 ± 1.39	6.47 ± 0.56			
miR-T2-3p		0.83 ± 0.87			2.80 ± 0.67	2.48 ± 1.26			
miR-T3-3p		9.89 ± 4.67			36.29 ± 8.78	14.15 ± 8.30			**2.71** ± 4.70*
miR-T3/T4-5p		14.17 ± 1.46	0.34 ± 0.35		27.02 ± 4.75	9.73 ± 2.65	*0.17* ± *0.24*		
miR-T4-3p		8.52 ± 1.75			19.69 ± 5.15	5.02 ± 2.13			
miR-VP-3p					0.28 ± 0.41	**0.08** ± 0.14*			

Note: Data are presented as the mean ±SD of three technical replicates. Values in bold font indicate amplification in only one of three replicates. Blank cells indicate that the corresponding miRNA was not detected. The corresponding EBOV load for each individual is given in parentheses below the patient designator. Values in italicized font denote miRNA concentration higher than viral load.

miRNAs were detected in samples collected 2–18 days post-symptom onset, and samples with the highest viral load (G5 and G6) were positive for all miRNAs assayed. Similarly, all miRNAs except miR-VP-3p were detected in sample G2, which had the third-highest viral concentration. miR-1-5p was the most abundant miRNA (mean concentration 99.43 ± 142.16 fM), followed by miR-T3-3p (mean 15.76 ± 14.48 fM), miR-1-3p (mean 15.51 ± 9.00 fM) and miR-T3/T4-5p (mean 10.28 ± 11.14 fM). miR-T2-3p, miR-2-3p, and miR-VP-3p were at the lowest concentrations, comparable to results from the NHP and mouse cohorts.

miR-1-5p was detected in the most samples (7 patients), followed by miR-T3/T4-5p and miR-T1-3p (5 patients). While viral load was quantifiable in all samples tested, miRNAs were not amplified in samples G7, G10–G13 and G15, when circulating virus levels dropped to less than 0.2 fM (<150 PFU/mL). However, miR-1-5p was detected in sample G9 (viral load = 0.161 fM) and miR-T3-3p amplified in one technical replicate of sample G14 (viral load = 0.104 fM, 18 days after symptom onset).

The majority of miRNAs were at lower concentrations compared to circulating EBOV, but miR-1-5p was 40-fold and 1.3-fold higher than viral glycoprotein in samples G8 and G9, respectively. Similarly, miR-T3-3p was present at a concentration 26 times higher than virus in sample G14, miR-T3/T4-5p was 5-fold higher in sample G8, and miR-1-3p was 1.8-fold higher in sample G4. In these patient samples with increased miRNA/viral RNA ratios, EBOV load was low (<13 fM).

### Comparison of EBOV miRNA profiles during mouse, macaque, and human disease

**Figure 4:**
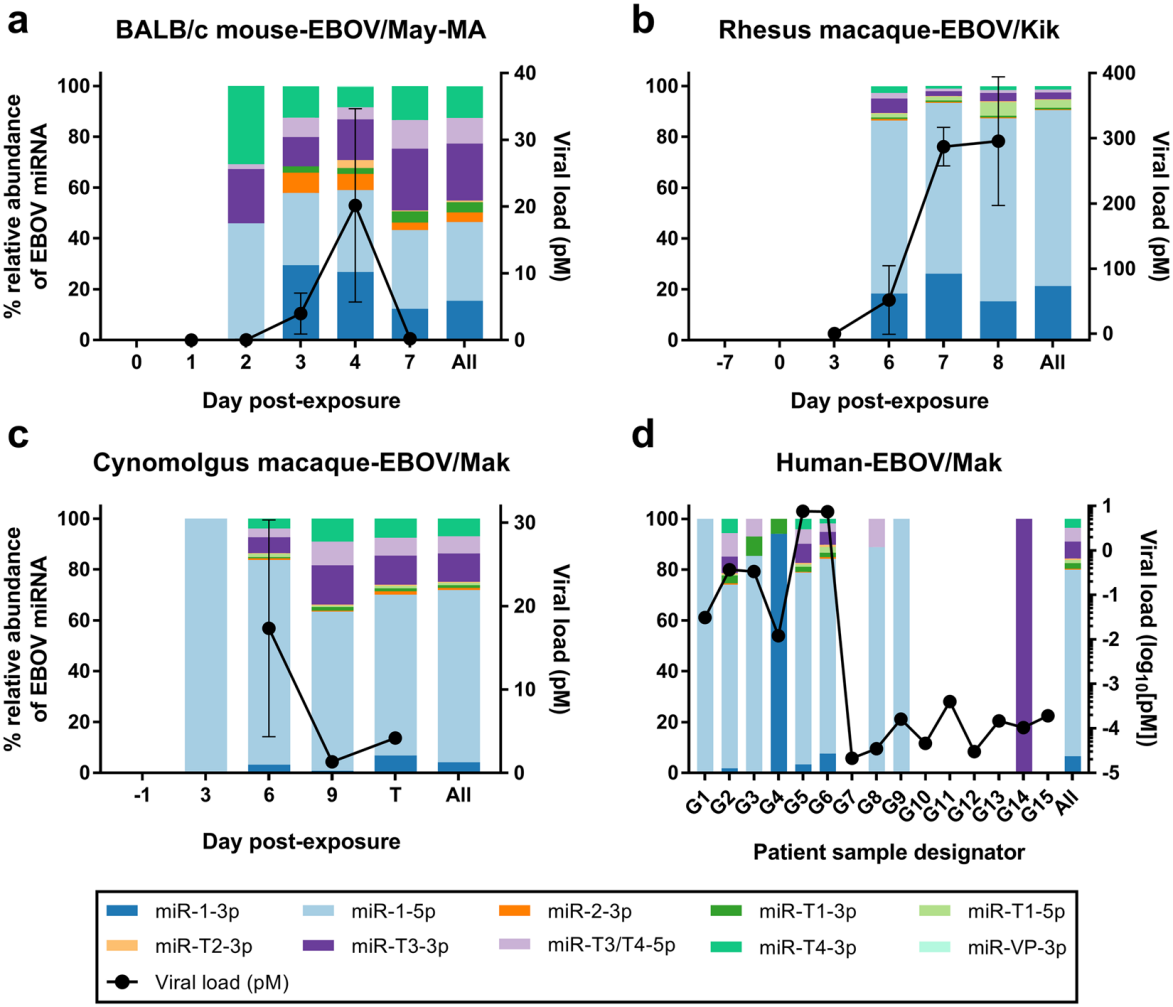
EBOV miRNA distributions are similar in different disease models. The relative abundance of each EBOV miRNA species detected was plotted as a percentage of the total EBOV miRNA content per day in the (**a**) BALB/c mouse model infected with mouse-adapted EBOV/Mayinga, (**b**) rhesus macaque model infected with EBOV/Kikwit, (**c**) cynomolgus macaque model infected with EBOV/Makona, and (**d**) human samples from the EBOV/Makona outbreak in 2014. In the animal models, samples were grouped by day post-exposure, whereas human samples are shown individually and arranged by day post-symptom onset. The aggregated miRNA distributions for each model are given in the last bar for each graph. EBOV viral loads (mean ± SD of biological replicates, where applicable, assayed in technical triplicate) are shown for comparative purposes, and filled circles represent a positive PCR result for the viral glycoprotein sequence.

To better visualize EBOV miRNA trends in these different disease models, we plotted the relative abundance of each miRNA species as a percentage of the total EBOV miRNA content per day (Fig. 4). In the mouse model, miR-1-5p was the most abundant on all days, representing an average of 31.0% of the total over the disease course, followed by miR-T3-3p and miR-1-3p at 22.4% and 15.4%, respectively. In rhesus macaques, miR-1-5p dominated the total EBOV miRNA content at 69.1% over all days, followed by miR-1-3p at 21.3% and miR-T3-3p at 2.7%. miR-1-5p was also the most represented miRNA in the cynomolgus macaque EBOV/Makona infection at an average of 67.7% of the total over all days, followed by miR-T3-3p (11.2%), miR-T4-3p (6.9%), miR-T3/T4-5p (6.7%), and miR-1-3p (4.2%). In the human cohort, a similar pattern of miRNA abundances emerged, with miR-1-5p garnering an average of 73.4% over all days, and trailed by miR-T3-3p (6.6%), miR-1-3p (6.5%), and miR-T3/T4-3p (5.4%).

In all EBOV infection models, miR-1-5p was the most abundant miRNA, comprising ~70% of the EBOV miRNAs detected in two macaque species and in humans. While still the main miRNA present in mouse EVD, miR-1-5p abundance was proportionally lower and closer to the next most-abundant miRNAs miR-T3-3p and miR-1-3p. These latter two are also among the top miRNAs detected in all EBOV models, along with miR-T3/T4-5p. No significant correlations were observed between viral load and individual miRNA abundances in any of the infection cohorts.

### Viral miRNA target prediction and gene set analysis

To determine whether viral miRNAs elicit similar responses in the species tested, we predicted gene targets using TargetScan Custom v5.2, which uses the miRNA seed sequence to calculate binding probabilities in various species; here, we searched for hits against human, rhesus macaque, and mouse. Input of the miR-T1-3p seed sequence revealed that it is identical to that of the endogenous mouse miRNA mmu-miR-470. None of the other EBOV miRNA seed sequences mapped to host-encoded miRNAs using this method.

**Figure 5:**
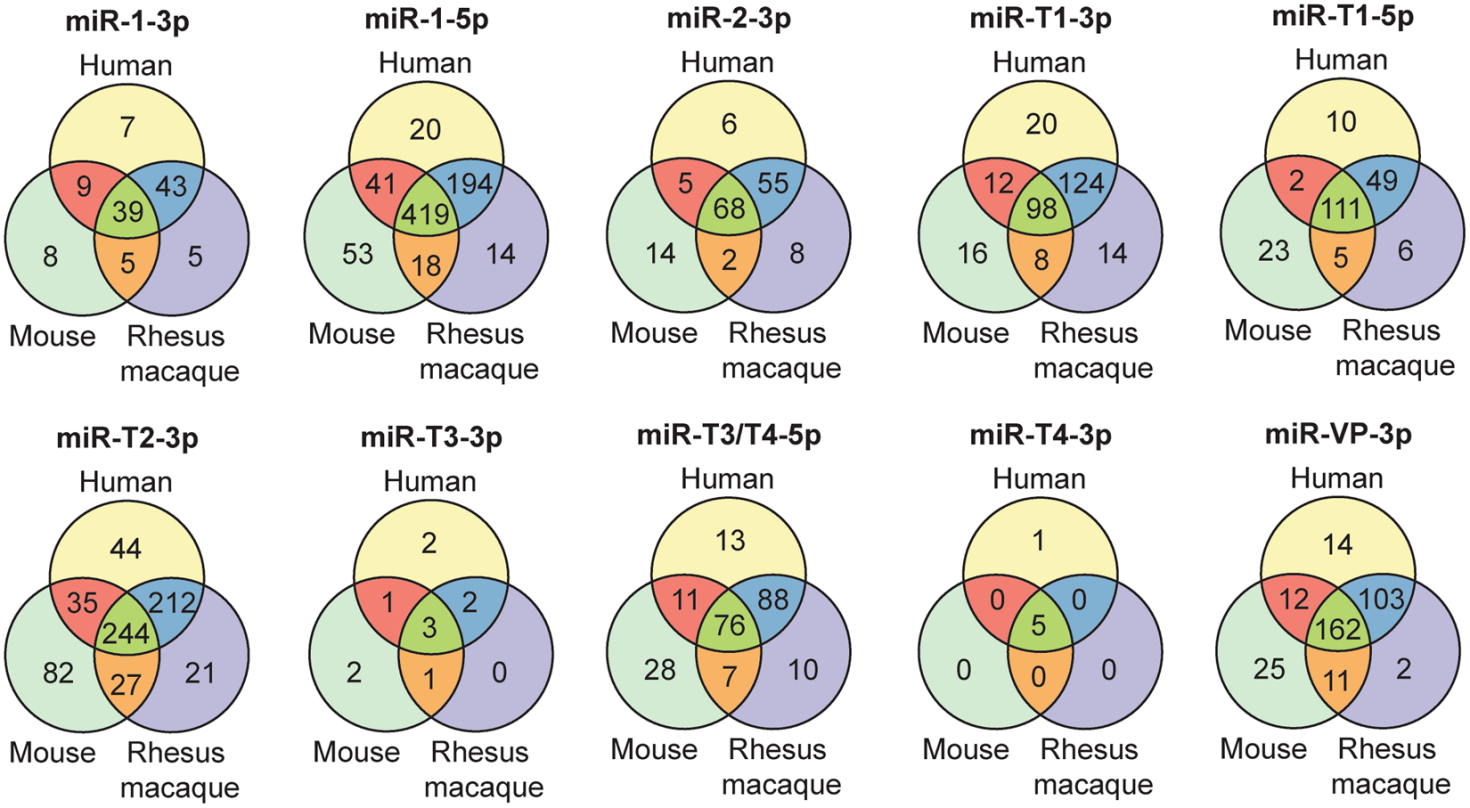
EBOV-encoded miRNAs target a conserved set of genes in human, rhesus macaque, and mouse. A majority of the EBOV-encoded miRNA target genes are conserved in humans, rhesus macaques, and mice. Venn diagrams denote the overlaps in the genes predicted using viral miRNA seed sequences input into TargetScan Custom v5.2 for each species.

We found that each EBOV-encoded miRNA targeted many of the same genes, regardless of species (Fig. 5). The intersection of genes comprised an average of 52%, 57%, and 73% of the total number of human, rhesus macaque, and mouse genes predicted, respectively (data not shown). The top three miRNAs with the most predicted gene targets were miR-1-5p, miR-T2-3p, and miR-VP-3p.

We then investigated the potential regulatory functions of the top 3 most-abundant miRNAs in all species, miR-1-3p, miR-1-5p, and miR-T3-3p. As human miRNA-mRNA interactions are currently better annotated through experimental validation^20^, we used the human target gene predictions as input into ToppFun^21^ to explore possible effects on biological function through enrichment analyses. The top three viral miRNAs appear to regulate divergent biological processes (Table 5). Genes targeted by miR-1-3p are involved in clathrin-mediated endocytosis, while those regulated by miR-1-5p participate in c-MET signaling. miR-T3-3p target genes, meanwhile, are implicated in activin antagonism.

**Table 5:** Human biological pathways potentially regulated by the most abundant miRNA target genes.

miRNA	Overrepresented pathway	P-value
miR-1-3p	Cargo recognition for clathrin-mediated endocytosis	1.12E-05
	Clathrin-mediated endocytosis	7.06E-05
	Disease	1.02E-04
miR-1-5p	MET activates RAS signaling	2.57E-08
	Signaling pathways regulating pluripotency of stem cells	7.96E-07
	Hippo signaling pathway	3.81E-06
	Signaling by MET	7.12E-06
	Signaling events mediated by Hepatocyte Growth Factor Receptor (c-Met)	1.12E-05
miR-T3-3p	Antagonism of Activin by Follistatin	1.61E-03

## Discussion

In this work, we demonstrated that putative EBOV-encoded miRNAs are detectable in circulation in different infection models (mouse, rhesus macaque, cynomolgus macaque, and human) of various EBOV variants (EBOV/Mayinga-MA, EBOV/Kikwit, and EBOV/Makona). We established experimentally that predicted EBOV miRNAs are conserved in four mammalian species, in agreement with computational alignment to viral genomes.

EBOV miRNAs amplified in cell culture supernatants, with the most abundant miRNAs being miR-1-5p, miR-1-3p, and miR-T3-3p. However, despite standardizing viral input, differing copy numbers and miRNA levels were detected by PCR. Currently there are no clear relationships between EBOV in clinical samples, PFU/mL, and copies/mL, but estimates suggest that viral copies are 100- to 10,000-fold more abundant than infectious virus^22,23^. In this work, 1 PFU of TRIzol LS-inactivated stock virus corresponded to ~2800 viral copies of the RNA standard.

Ten of the twelve predicted EBOV miRNAs were detectable in NHP plasma from rhesus macaques challenged with EBOV/Kikwit, and in cynomolgus macaques injected with EBOV/Makona. miR-1-5p, miR-1-3p, and miR-T3-3p were among the top miRNAs detected in both groups, with lower miRNA-to-virus ratios for the EBOV/Makona group; for example, miR-1-5p concentrations were ~257-fold lower than virus in the EBOV/Kikwit group compared to ~54-fold lower in the EBOV/Makona cohort. A majority of miRNA levels also increased over the disease course despite dissimilar patterns in EBOV abundance. These results could indicate that miRNAs are actively secreted^15^ or are released during cell death^24^ as EVD progresses. Intriguingly, miR-1-5p was detected in one presymptomatic, virus-negative (day 3) cynomolgus macaque infected with EBOV/Makona (out of three NHPs assayed). No miRNAs were detected presymptomatically in the EBOV/Kikwit rhesus macaque group, despite higher overall viral and miRNA concentrations in that cohort. However, for both NHP studies no blood was collected between day 3 (infected but presymptomatic and virus-negative) and day 6, when animals were typically acutely symptomatic and presented high EBOV titers. Daily blood collection in these models may resolve whether EBOV miRNAs can be reliable markers of EVD prior to the detection of circulating virus. One report of the amplification of miR-VP-3p in virus-negative but symptomatic human cases found this miRNA in exosomes, and postulated that it is actively secreted by the virus prior to positive viral PCR^15^. If all EBOV miRNAs are secreted in exosomes, these may be accessible targets before viral load is detectable and prior to the onset of symptoms.

We investigated the potential of EBOV miRNAs as presymptomatic markers of disease in the mouse model, as daily terminal bleeds were collected in this study. Similar to the NHP groups, miR-1-5p, miR-1-3p, and miR-T3-3p were also present at the highest levels in this cohort, albeit with more balanced proportions of these top miRNAs relative to the total miRNA amount. The temporal patterns of EBOV miRNA abundance also exhibited varied trends, with the majority of miRNAs at low levels until day 7. Again, elevated miRNA levels could be products of miRNA release due to cell apoptosis or necrosis from EVD. miR-T1-5p, with a mismatch in the miRNA seed sequence, showed low levels of amplification at high Cq values (>35), with a different melt curve compared to the actual target. This suggests either low levels of amplification and/or a lack of predicted miR-T1-5p in mouse samples. Interestingly, the G-to-A substitution in the miR-T1-5p seed sequence corresponds to one of two key mutations in the viral genome that confers virulence to the mouse-adapted EBOV strain, likely by enabling the virus to evade the host antiviral type I interferon response^25^.

In this mouse model, we observed presymptomatic (day 2) detection of four miRNAs in three different mice, while viral glycoprotein amplified as early as day 1 post-infection. miRNA concentrations on day 7 rivaled those of viral load, with miR-1-5p only ~1.65 times lower than corresponding EBOV titer. These results indicate that viral miRNAs may also be feasible diagnostic targets of EVD for mouse-adapted EBOV infection.

Finally, we assayed serum/plasma from human EVD cases in Sierra Leone, and verified the presence of the ten predicted EBOV miRNAs in these samples. Similar to the NHP and mouse groups, miR-1-5p, miR-T3-3p, and miR-1-3p presented at the highest concentrations. The relative abundances of the miRNAs in this cohort mirrored the NHP groups, with miR-1-5p dominating the viral miRNA landscape. Paralleling the cynomolgus macaque cohort, miRNAs were relatively high compared to viral load, with the top 3 miRNAs at ~200 × lower. The observation that viral miRNA concentrations were reduced by orders of magnitude compared to viral genomes is not unexpected since Rouha *et al*. reported that viral copies of tick-borne encephalitis virus were ~140 × greater than miRNA copies *in vitro* at 48 h post-infection^7^. Unlike the animal models, however, in human infection we observed miRNA levels higher than circulating virus in samples where viral load was relatively low. Again, this indicates that the most abundant viral miRNAs may be used for EVD diagnosis.

A recent study reported that miR-VP-3p was detectable in 27 EBOV-positive patients and in 6 symptomatic patients 1-2 days prior to positive viral PCR^15^. Our findings on miR-VP-3p are in contrast to this study, where average EBOV load was 220.9 pM and the corresponding miR-VP-3p concentration was 205.3 fM (~1000-fold lower) by TaqMan miRNA assay^15^. In our human samples, EBOV/Makona viral loads were much lower and ranged from 2.1 × 10^−5^ to 7.64 pM (mean 1.06 pM), with average miRNA levels from the most abundant viral miRNA, miR-1-5p, at 99.43 fM (~11-fold lower). miR-VP-3p abundances for these same samples, as assayed by LNA-based primers, was reduced at 0.18 fM (almost 6000 times lower than viral load), and found only in 2 of 15 samples tested. miR-VP-3p was also the least abundant miRNA in the four EBOV-infected species tested here. The discrepancy in miR-VP-3p quantitation between our study and that of Chen *et al*.^15^ could be attributed to differences in the miRNA amplification chemistries used. We selected LNA-based primer sets because of the high sensitivity and selectivity compared to TaqMan-based assays^16–18^. This technology choice may account for differences in miR-VP-3p quantitation, as side-by-side comparisons of the two platforms showed that copy number estimates for the same samples are statistically significantly different^16^. For this work, we originally tested two Exiqon primer designs and the TaqMan Small RNA Assay for miR-VP-3p with the same synthetic miR-VP-3p sequence and found that the Exiqon primers performed slightly better (Supplementary Fig. S5). In light of this, and because the TaqMan miRNA assay requires a separate cDNA synthesis for each miRNA to be amplified, we pursued testing only with Exiqon primers.

Current understanding of viral transcription indicates that EBOV mRNAs are sequentially processed from the 3′ end, leading to an mRNA gradient with nucleoprotein (NP) transcripts being most abundant, followed by VP35, VP40, GP, VP24, and L transcripts^26^. Quantitation of EBOV-encoded miRNAs in all infected samples indeed showed that miRNAs which aligned to the NP sequence were more abundant than miR-T2-3p, miR-VP-3p, and miR-2-3p, which are found closer to the 5′ end of the genome. miR-1-3p and miR-1-5p, which were among the top miRNAs detected, lie in the intergenic region between VP30 and VP24. This region is a long untranscribed span that is critical for the expression of neighboring genes^27,28^ and may account for the increased quantities of these viral miRNAs.

Analysis of the distributions of viral miRNAs in the four species revealed that miR-1-5p was the predominant EBOV miRNA in all infection models, followed by miR-T3-3p and miR-1-3p. *In silico* prediction of viral miRNA binding partners showed that the majority of targeted genes are conserved in human, rhesus macaque, and mouse. This indicates that viral miRNAs likely regulate similar pathways in different species. Enriched pathways from human target gene lists predict that miR-1-3p, miR-1-5p, and miR-T3-3p exert control over different processes in EBOV pathogenesis. miR-1-3p putatively governs clathrin-mediated endocytosis, which is an entry mechanism used by EBOV^29,30^. miR-1-5p target genes, meanwhile, may control c-Met signaling. c-Met is a receptor tyrosine kinase (RTK) that triggers the PI3K signaling pathway to promote the infectivity of influenza A virus^31^. Similarly, EBOV cell entry requires early activation of the PI3K pathway, which is controlled by RTKs^32^, but these initial interactions have not yet been experimentally elucidated in EBOV infection. Pathway analysis also showed that miR-T3-3p may control activin antagonism by follistatin. Activin A is a member of the TGF-β family and is tightly regulated by its binding protein, follistatin, with both proteins expressed in hepatocytes. Activin A triggers a complex range of immunoregulatory functions and can be pro- or anti-inflammatory depending on disease and immune context^33^. While activin A and follistatin have not been explicitly studied in EVD, EBOV infection activates TGF-β signaling and protein production in hepatocytes, and inhibition of kinases in this pathway substantially decreased viral replication *in vitro*^34^. We should note that TargetScan predicted only a few target genes for miR-T3-3p. As this algorithm uses only the miRNA seed sequence to search for 3′ UTR binding partners, miRNA interactions such as mRNA binding strength and target site accessibility are unaccounted for in this model^35^.

Virus-encoded miRNAs are typically analogs of host miRNAs or specific to the virus. Analogs are miRNAs that share seed sequences (2-7 or 2–8 nt relative to the 5′ end), and virus-produced analogs can potentially control the hundreds of mRNAs that the host miRNA can bind. We found that miR-T1-3p shares the same seed sequence as the endogenous mouse miRNA mmu-miR-470, while miR-1-5p shares the same seed region as hsa-miR-155-5p^14^, indicating that these may function as host miRNA analogs. It is also noteworthy that the most abundant EBOV miRNAs in EBOV-infected samples regulate target genes *in vitro;* for instance, miR-1-5p experimentally downregulated importin-α5 in HEK293T cells^14^, while miR-T3-3p inhibited the expression of HDAC5 and RIPK (genes not originally predicted for this miRNA by TargetScan)^12^. EBOV VP24 targets importin-α5 to repress STAT1 signaling and counteract the host antiviral interferon response^36^. Meanwhile, RIPK is a central mediator of the NF-κB and TNF signaling pathways; EBOV miRNA silencing of these could suppress host immune defenses and enhance viral replication and spread. Taken together, these results strongly suggest that EBOV miRNAs may play an integral role in promoting viral infectivity and are potentially targetable disease elements. While our primary effort was to determine the diagnostic applicability of these virus-encoded miRNAs, further study of these abundant miRNAs may provide greater insight into EBOV replication, infectivity, and pathogenesis.

## Materials and Methods

### Candidate miRNA sequence alignments to EBOV genomes and prediction of miRNA seed sequence mismatch effects

Predicted pre-miRNA and mature miRNA sequences were aligned to GenBank reference sequence KM034562.1 (*Zaire ebolavirus* isolate Ebola virus/H.sapiens-wt/SLE/2014/Makona-G3686.1) using the BLAST-Like Alignment Tool (BLAT) of the UCSC Ebola Genome Browser^37^. The Genome Browser includes multiple alignments of 158 Ebola genomes from outbreaks spanning 1976–2014, as well as two *Marburgvirus* genomes for comparison. Candidate miRNAs were also used as input into the GenBank Nucleotide BLAST tool using default parameters for a short input sequence.

The effect of the single-base substitution in the seed sequence of miR-T1-5p was assessed using miRmut2GO^19^, which predicts the target genes and enriched functional annotations for the original and mutant miRNA sequences, and compares the similarity between the two groups of GO terms. It calculates a similarity score between 0 and 1 for the two miRNAs, with 0 indicating no similarity and 1 denoting functionally identical miRNAs.

### miRNA sequences and primers

miRNA sequences included in this study (12 miRNAs, Supplementary Table S1) were previously predicted and reported^11,12,14,15^. Synthetic miRNAs, as well as two LNA primer sets per target (custom miRCURY LNA™ Universal RT microRNA PCR Assays) were obtained from Exiqon, Inc. (Woburn, MA). Primer sets were designed using their custom qPCR primer web tool (https://www.exiqon.com/mirna-qpcr-designer), and design IDs for each set are given in Supplementary Table S2.

### NHP and human samples

#### Ethics statement and disclosures

Cynomolgus macaque, mouse, and rhesus macaque cell-free and whole blood samples were previously archived samples and not collected specifically for this study. Animal research was conducted under IACUC-approved animal protocols at the United States Army Medical Research Institute of Infectious Diseases (USAMRIID). These protocols complied with the Animal Welfare Act, Public Health Service Policy, and other Federal statutes and regulations relating to animals and experiments involving animals. The facility where this research was conducted is accredited by the Association for Assessment and Accreditation of Laboratory Animal Care, International and adheres to principles stated in the Guide for the Care and Use of Laboratory Animals^38^. Post-exposure, animals were evaluated daily for signs of illness. Following development of clinical signs, animals were checked multiple times daily. Institute scoring criteria were used to determine timing of humane euthanasia under anesthesia^39^. All animal experiments were conducted in USAMRIID’s ABSL-4 laboratory.

Research on human subjects was conducted in compliance with United States Department of Defense, federal, and state statutes and regulations relating to the protection of human subjects, and adheres to principles identified in the Belmont Report^40^. All data and human subjects research were gathered and conducted for this publication under a USAMRIID Institutional Review Board-approved protocol.

De-identified human serum and plasma samples were used in this study. These samples were determined by the USAMRIID Office of Human Use and Ethics to be Not Human Subject Research. All samples were collected and de-identified in Sierra Leone at Kenema Government Hospital. The samples had indirect identifiers upon receipt. None of the samples were collected specifically for this study. Samples were collected during the outbreak to perform emergency diagnostics. In this context, the need for written informed consent was waived by the Ministry of Health and Sanitation of the Republic of Sierra Leone.

The opinions, interpretations, conclusions, and recommendations contained herein are those of the authors and are not necessarily endorsed by the United States Army.

#### Rhesus macaques exposed to EBOV/Kikwit

Plasma samples used in this work were archived samples not collected for the purpose of this study, but were from the *in vivo* characterization of a challenge material working stock (stock propagation details in Supplementary Methods). Three male and three female adult rhesus macaques (*Macaca mulatta*) of Chinese origin were exposed to a target dose of 1000 plaque-forming units (PFU) of Ebola virus/H.sapiens-tc/COD/1995/Kikwit-9510621 (EBOV/Kikwit) in a head-only aerosol chamber within a biosafety level 4 (BSL-4) laboratory. Blood samples were collected prior to virus exposure at day -7 and day 0 relative to infection. Additional samples were collected at post-exposure days 3, 6, and at euthanasia (days 7 and 8). Whole blood was collected in K_2_ EDTA tubes and centrifuged to yield plasma. Plasma samples were mixed with 3 volumes of TRIzol LS (Life Technologies, Grand Island, NY) and stored at −80 °C. In this work, samples from the six NHPs exposed to aerosolized EBOV/Kikwit were assayed for EBOV miRNAs.

#### Cynomolgus macaques exposed to EBOV/Makona

Plasma samples used in this work were archived samples not collected for the purpose of this study, but for a larger study (n = 15) investigating NHP infection with EBOV/Makona at different doses and routes of infection^41^. Chinese-origin cynomolgus macaques *(Macaca fascicularis*) were used for this study (age range: 3–9 years). Prior to initiation of the study, the animals were found to be negative for Herpes B Virus, SRV-1, -2, and -3, SIV, and STLV-1 and negative for EBOV-specific antibodies. NHPs were exposed to EBOV/Makona; this variant was produced from a master stock that was made from an isolate from serum of a 2014 Sierra Leone fatal human case (SL 3864.1, GenBank accession #KR013754.1, additional information in Supplementary Methods^41^). Samples used in this study were from three male cynomolgus macaques infected intramuscularly with 1000 PFU of virus. Blood samples were collected at various time points for isolation of plasma and serum for analysis of chemistry and hematological parameters, detection of viral genomes by RT-qPCR, and assessment of antibody responses.

#### BALB/c mice exposed to mouse-adapted EBOV/Mayinga

BALB/c mice (7–9 weeks old) were infected with 100 PFU mouse-adapted EBOV/Mayinga (EBOV/May-MA) by intraperitoneal injection. Additional details on viral stock propagation are available in Supplementary Methods^42^. Five naïve mice served as controls and were euthanized at day 0 relative to virus exposure. In this serial sacrifice study design, five mice were scheduled for euthanasia at 1, 2, 3, 4, and 7 days post-exposure to collect whole blood and serum. Mice were deeply anesthetized before exsanguination by intercardiac puncture, and whole blood (300 µL) was immediately added to a prepared solution of 300 µL of water and 1.8 mL (3 volumes) TRIzol LS.

In this work, BALB/c mouse whole blood samples (n = 17) were investigated for EBOV miRNAs. Mouse samples from three terminal bleeds were collected on days 0, 1, 2, 3, and 4 post-infection, and samples from two mice were obtained from day 7 terminal bleeds.

#### Human samples from 2014 EBOV/Makona outbreak

Fifteen human clinical serum or plasma samples^6^ were obtained from Kenema Government Hospital between June and August 2014 during the EBOV/Makona outbreak in the Republic of Sierra Leone, West Africa. The patient set consists of seven males and seven females, with ages ranging between 17 and 58 years (median age 39 years). Two samples from the same subject, taken 4 days apart, were included in this set. Patients were classified as EBOV-positive using fielded diagnostic assays^43^. Date of symptom onset was recorded based on patient self-reporting. Samples were collected during the outbreak to perform emergency diagnostics, and were inactivated with three volumes of TRIzol LS prior to processing, storage, and shipment. All archived human samples were further processed at a BSL-2 laboratory at USAMRIID.

### Total RNA extraction

Total RNA extractions from EBOV cell culture supernatants and mouse, NHP and human samples mixed with 3 volumes of TRIzol LS were performed using a previously optimized spin column-based method with the addition of glycogen and synthetic RNA spike-ins^44^. For NHP and human samples, archived aliquots of 50 μL plasma were processed with either the miRNeasy Mini Kit (50 μL elution), or with the miRNeasy Serum/Plasma Kit (12 µL elution). Mouse whole blood samples (175 µL) were extracted with the miRNeasy Mini Kit (50 µL elution). All kits were from QIAGEN, Inc. (Valencia, CA).

### Viral load determination using RT-qPCR

Viral loads in samples were determined with RT-qPCR using an internally validated TaqMan probe assay targeting an 80-bp sequence in the glycoprotein^45^ that amplifies both EBOV/Kikwit and EBOV/Makona^46^. A standard curve was created from serial dilutions of the EBOV/Kikwit challenge stock (provided as PFU/mL based on plaque assay) extracted identically to plasma samples, and Cq values were calculated using the second derivative maximum method implemented in the integrated Roche LightCycler 480 software version 1.5.1 (Roche, San Francisco, CA).

Viral copy numbers (given as copies/mL) were determined using the same RT-qPCR assay run with synthetic RNA spanning the glycoprotein target sequence, as previously described^45^.

### cDNA synthesis and miRNA profiling

Synthetic miRNA and extracted total RNA was reverse-transcribed with the cDNA Synthesis Kit II (Exiqon, Inc.) using 2 μL RNA for each 10 μL cDNA mix. cDNA samples were diluted 40× (per manufacturer’s instructions) and amplified on a LightCycler 480 II. For quantitation of candidate miRNAs, synthetic miRNAs were serially diluted from 50 to 0.000005 pM (8 dilutions) and used to construct standard curves for interpolation/extrapolation and to evaluate primer set performance (Supplementary Fig. S4).

### LNA-based miRNA primer testing and downselection

Synthetic miRNA targets and a no-template cDNA synthesis control were used to test the amplification efficiencies and specificities of the LNA-based primes. Next, to determine whether putative miRNAs are present in EBOV cell culture supernatant, primer sets were tested with total RNA from serially-diluted EBOV/Kikwit viral stock (10^6^–10^0^ PFU/mL). We tested for putative miRNAs in other EBOV variants using RNA from 10^6^ PFU/mL of EBOV/Gabon and EBOV/Makona. Specificity was also tested by using RNA from 10^6^ PFU/mL of Marburgvirus Musoke (MARV/Musoke) variant as input. Candidate miRNAs were assayed in NHP samples with known EBOV titer from the aerosol-exposed EBOV/Kikwit infection cohort.

### miRNA target prediction and gene set analysis

We used TargetScan Custom v5.2 to predict viral miRNA gene targets in human, rhesus macaque, and mouse using the miRNA seed sequence (2–8 nt from the 5′ end) as input^47^. We then used ToppFun^21^ with default parameters (hypergeometric distribution with Bonferroni correction, P-value < 0.05) on predicted human target genes for the three most abundant miRNAs (miR-1-5p, miR-1-3p, and miR-T3-3p) to perform gene set functional enrichment.

## Electronic supplementary material

Supplementary information
