# Supplementary material for: Virus-encoded miRNAs in Ebola virus disease
Source: Sci Rep. 2018 Apr 24;8:6480. doi: 10.1038/s41598-018-23916-z (PMC5915558; doi:10.1038/s41598-018-23916-z)
Supplement: Supplementary file 1 — Supplementary information [file 41598_2018_23916_MOESM1_ESM.docx]

**Supplementary Information: Virus-encoded miRNAs in Ebola virus disease**

Janice Duy^a^, Anna N. Honko^b^, Louis A. Altamura^a^, Sandra L. Bixler^b^, Suzanne Wollen-Roberts^b^, Nadia Wauquier^c+^, Aileen O’Hearn^a^, Eric M. Mucker^b^, Joshua C. Johnson^b^, Joshua D. Shamblin^b^, Justine Zelko^b^, Miriam A. Botto^b^, James Bangura^c^, Moinya Coomber^c^, M. Louise Pitt^b^, Jean-Paul Gonzalez^d#^, Randal J. Schoepp^a^, Arthur J. Goff^b^, Timothy D. Minogue^a*^

Diagnostic Systems Division, U.S. Army Medical Research Institute of Infectious Diseases, Fort Detrick, Frederick, MD, USA^a^; Virology Division, U.S. Army Medical Institute of Infectious Diseases, Fort Detrick, Frederick, MD, USA^b^; Metabiota, Kenema, Sierra Leone^c^; Metabiota, Washington, DC, USA^d^

^*^Address correspondence to Timothy D. Minogue, [timothy.d.minogue.civ@mail.mil](mailto:timothy.d.minogue.civ@mail.mil)

^+^Current address: MRIGlobal – Global Health Surveillance and Diagnostics, Gaithersburg, MD, USA

^#^Current address: Center of Excellence for Emerging & Zoonotic Animal Disease, Kansas State University, Manhattan, KS, USA

**Supplementary Methods: Viral stock propagation**

***EBOV/Kikwit***

Vero E6 cells (ATCC, 75% confluent) with MEM Alpha containing GlutaMax-I and 10% heat inactivated fetal bovine serum were infected with Ebola virus/H.sapiens-tc/COD/1995/Kikwit-9510621 at MOI = 0.01. No antibiotics were used. Supernatant was collected from cultures at 3+ cytopathic effect (four days post-inoculation). The supernatant virus was clarified by centrifugation (3,000 g x 10 min). One ml of clarified supernatant was dispensed into cryovials. Product was maintained at or below -70ºC until used.

***EBOV/Makona (methods recently published as part of the original study in Speranza et al. (2017))***

A working seed bank of the isolate Ebola virus/H.sapiens/wt/SLE/2014/Makona-G3864.1 (EBOV/Mak) was produced from a master stock that was made from an isolate from serum of a 2014 Sierra Leone fatal human case (SL10 3864.1). The working stock was produced by infecting Vero E6 cells at a multiplicity of infection of 0.001 in T175 flasks. Virus was diluted and propagated in Eagle’s Minimum Essential Medium (EMEM) supplemented with 2 % [vol/vol] heat-inactivated fetal bovine serum (ΔFBS; HyClone), 2 mM L-glutamine (HyClone), 100 IU penicillin (Cellgro), 1000 μg/mL streptomycin (Cellgro), and 1.25 μg/ml amphotericin B (ThermoFisher Scientific). On day 9 post-inoculation, the media was harvested, an additional 8 % ΔFBS added after clarified by centrifugation, and aliquoted into 1-ml single-use cryovials. The preparation was a Vero E6 passage 2 stock and contained an average of 4.02x10^6^ plaque-forming units per ml (PFU/mL) of infectious virus. This stock was evaluated for sterility on trypticase soy broth and chocolate agar plates and tested for mycoplasma and endotoxin levels using Lonza’s Endpoint Chromogenic Limulus Amebocyte Lysate (LAL) test and MycoAlert biochemical assay. The stock was determined to have no detectable mycoplasma, endotoxin or adventitious agents based on the assays and techniques used. Total RNA isolated from this stock was sequenced and tested in a number of real-time reverse-transcription polymerase chain reaction (RT-qPCR) assays to include those specific for each filovirus. No known contaminates were detected when sequencing the stock, and identity was confirmed by real-time reverse transcription quantitative real-time PCR (RT-qPCR). By electron microscopy, virions exhibited morphological characteristics typical of filoviruses in that they appeared as either long filamentous or rod forms, sewing needle shaped, or in shorter mace-shaped configurations. Particles varied in length with a uniform diameter of approximately 80 nm.

***Mouse-adapted EBOV/Mayinga (from Herbert et al. (2015))***

Vero E6 cells (ATCC) were maintained in Eagle’s minimal essential medium (EMEM) supplemented with 5% heat-inactivated fetal bovine serum (ΔFBS) and gentamicin (50 µg/ml) at 37°C, 5% CO_2_, and 80% humidity. HUVECs (ATCC) were maintained in endothelial cell Basal Medium-2 (Lonza) supplemented with 5% ΔFBS and penicillin-streptomycin (100 U; 100 µg/ml) at 37°C, 5% CO_2_, and 80% humidity. Mouse-adapted EBOV was used for all mouse challenge studies.

**Supplementary Table S1**. **Putative Ebola virus pre-miRNAs and mature miRNAs aligned to viral genomes in UCSC Ebola Genome Browser.** Alignment was performed to GenBank entry KM034562.1 (Zaire ebolavirus isolate Ebola virus/H.sapiens-wt/SLE/2014/Makona-G3686.1, EBOV/Mak).

| Pre-miRNA/miRNA | Sequence (5’ to 3’) | **Genome position on EBOV/Mak KM034562.1** | Coding region | Reference |
| --- | --- | --- | --- | --- |
| EBOV-pre-miR-1 (Liang) | AUAGAACGAGGAAGAUUAAGAAAAAGUCCAUAAUGCUGGGGAGGCAAUCCUUGCCACCAUAGGACUUUUUCAAUUCCUCUAUUUUAU | NA | NA | (Liang et al. 2014) |
| EBOV-pre-miR-2 | AUAAACCUCUUGAUUUCGGGACCAUUGCACUAUCCUUAGCAGUUCCUCAGGUAUUGGGUGGAUUAUCCUUCCUAAAUCCAGAAAAGUGC | NA | NA | Liang et al. (2014) |
| EBOV-pre-miR-T1 | UUGAUUUUCAAGAGAGUGCGGACAGUUUCCUUCUCAUGCUUUGUCUUCAUCAUGCGUACCAAGGAGAUUACAAACUUUUCUUGGAAAGUGGCGCAGUCAAGUAUUUGGAAGGGCACGGGUUCCGUUUUGAAGUCAA | 660-795 | NP | Teng et al. (2015) |
| EBOV-pre-miR-T2 | AACCAAAAAUGAUGAAGAUUAAGAAAAACCUACCUCGACUGAGAGAGUGUUUUUUCAUUAACCUUCAUCUUGUAAACGUU | 4380-4459 | Pre-VP40 | Teng et al. (2015) |
| EBOV-pre-miR-T3/T4 | GGAUGACGCCGAGUCUCACUGAAUCUGACAUGGAUUACCACAAGAUCUUGACAGCAGGUCUGUCCGUUCAACAGGGGAUUGUUCGGCAAAGAGUCAUCC | 495-593 | NP | Teng et al. (2015) |
| EBOV-pre-miR-VP | GCUGUAGGUCUUUUGAUCAGCGACACCUAGAGGAAGCCAAAUUGGAAUUUGCUUCAUUAGCACUUUGGGGCCCGAUAUGACAUUCACCAU | 4710-4799 (minus strand) | VP40 | Chen et al. (2016) |
| EBOV-pre-miR-1 | UGCUAAACUAAUGAUGAAGAUUAAUGCGGAGGUCUGAUAAGAAUAAACCUUAUUAUUCAGAUUAGGCCCCAAGAGGCAUUCUUCAUCUCCUUUUAGCA | 9874-9970 | Between VP30 and VP24 | Liu et al. (2016) |
| EBOV-miR-1-3p (Liang) | GCCACCAUAGGACUUUUUCAAU | NA | NA | Liang et al. (2014) |
| EBOV-miR-1-5p (Liang) | AAAAAGUCCAUAAUGCUGGGGA | NA | NA | Liang et al. (2014) |
| EBOV-miR-2-3p | UUAUCCUUCUUGAAUCCUGAGA | 14314-14335 | L | Liang et al. (2014) |
| EBOV-miR-T1-3p | UUCUUGGAAAGUGGCGCAGUCA | 737-758 | NP | Teng et al. (2015) |
| EBOV-miR-T1-5p | GGACAGUUUCCUUCUCAUGCUU | 679-700 | NP | Teng et al. (2015) |
| EBOV-miR-T2-3p | UGUUUUUUCAUUAACCUUCAUC | 4427-4448 | VP40 | Teng et al. (2015) |
| EBOV-miR-T3-3p | UGUCCGUUCAACAGGGGAUUGU | 555-576 | NP | Teng et al. (2015) |
| EBOV-miR-T3-5p/T4-5p | AUCUUGACAGCAGGUCUGUCCG | 539-560 | NP | Teng et al. (2015) |
| EBOV-miR-T4-3p | CUGUCCGUUCAACAGGGGGUUG | 554-575 | NP |  |
| EBOV-miR-VP-3p | UGCUUCAUUAGCACUUUGGGGC | 4729-4750  (minus strand) | VP40 | Chen et al. (2016) |
| EBOV-miR-1-3p | AGAUUAGGCCCCAAGAGGCAUU | 9931-9952 | Between VP30 and VP24 | Liu et al. (2016) |
| EBOV-miR-1-5p | AUUAAUGCGGAGGUCUGAUAAG | 9874-9970 | Between VP30 and VP24 | Liu et al. (2016) |

Supplementary Table S2. Exiqon Design IDs for custom LNA-based primer sets for amplification of putative Ebola virus miRNAs.

| **Primer name** | **Exiqon Design ID** |
| --- | --- |
| miR-1-3p-1 (Liang) | 514656-1 |
| miR-1-3p-2 (Liang) | 514656-2 |
| miR-1-5p-1 (Liang) | 514652-1 |
| miR-1-5p-2 (Liang) | 514652-2 |
| miR-1-3p-1 | 659009-1 |
| miR-1-3p-2 | 659009-2 |
| miR-1-5p-1 | 659005-1 |
| miR-1-5p-2 | 659005-2 |
| miR-2-3p-1 | 514660-1 |
| miR-2-3p-2 | 514660-2 |
| miR-T1-3p-1 | 514631-1 |
| miR-T1-3p-2 | 514631-2 |
| miR-T1-5p-1 | 514627-1 |
| miR-T1-5p-2 | 514627-2 |
| miR-T2-3p-1 | 514636-1 |
| miR-T2-3p-2 | 514636-2 |
| miR-T3-3p-1 | 514644-1 |
| miR-T3-3p-2 | 514644-2 |
| miR-T3-T4-5p-1 | 514640-1 |
| miR-T3-T4-5p-2 | 514640-2 |
| miR-T4-3p-1 | 514648-1 |
| miR-T4-3p-2 | 514648-2 |
| miR-VP-3p-1 | 592886-1 |
| miR-VP-3p-2 | 592886-2 |

Supplementary Table S3. EBOV miRNAs were detected in viral cell culture supernatants at differing concentrations. Total RNA from 10^6^ PFU of each EBOV variant tested was assayed for putative viral miRNAs using two LNA-based primer sets. miRNA concentrations are given as the mean ± SD of technical triplicates, and gray-shaded boxes denote extrapolated concentrations below the standard curve limit of quantification. ND = not detected.

| **miRNA primer set** | **miRNA concentration in cell culture (fM)** | | | |
| --- | --- | --- | --- | --- |
|  | **10^6^ PFU/mL EBOV/Kikwit** | **10^6^ PFU/mL EBOV/Gabon** | **10^6^ PFU/mL EBOV/Makona** | **10^6^ PFU/mL MARV/Musoke** |
| miR-1-3p-1 | 1.493 ± 0.268 | 0.011 ± 0.017 | 0.005 ± 0.007 | ND |
| miR-1-3p-2 | 1.835 ± 0.24 | 0.013 ±0.012 | 0.006 ± 0.011 | ND |
| miR-1-5p-1 | 0.024 ± 0.003 | 0.011 ± 0.001 | 0.009 ± 0.0003 | ND |
| miR-1-5p-2 | 0.013 ± 0.0001 | 0.006 ± 0.0005 | 0.004 ± 0.003 | ND |
| miR-2-3p-1 | 0.003 ± 0.002 | ND | 0.001 ± 0.0006 | ND |
| miR-2-3p-2 | 0.003 ± 0.003 | 0.0002 ± 0.0002 | 0.0005 ± 0.0005 | ND |
| miR-T1-3p-1 | 0.096 ± 0.056 | 0.004 ± 0.003 | 0.007 ±0.006 | ND |
| miR-T1-3p-2 | 0.111 ± 0.04 | 0.007 ± 0.007 | 0.002 ± 0.002 | ND |
| miR-T1-5p-1 | 0.0815 ± 0.006 | 0.099 ± 0.012 | 0.037 ± 0.024 | ND |
| miR-T1-5p-2 | 0.192 ± 0.143 | 0.031 ± 0.032 | 0.018 ± 0.025 | ND |
| miR-T2-3p-1 | 0.002 ± 0.001 | 0.002 ± 0.0002 | 0.0001 ± 0.0002 | ND |
| miR-T2-3p-2 | 0.001 ± 0.0003 | ND | 3.43E-05 ± 5.95E-05 | ND |
| miR-T3-3p-1 | 0.008 ± 0.006 | 0.001 ± 0.002 | 0.001 ± 0.002 | ND |
| miR-T3-3p-2 | 0.007 ± 0.002 | 0.0008 ± 0.0008 | 0.0002 ± 0.0002 | 0.0003 |
| miR-T3/T4-5p-1 | 0.002 ± 0.0008 | 0.0008 ± 0.0007 | ND | ND |
| miR-T3/T4-5p-2 | 0.001 ± 0.0003 | 0.0001 ± 0.0002 | ND | ND |
| miR-T4-3p-1 | 0.002 ± 0.001 | 0.0002 ± 3.32E-20 | 0.0005 ± 0.0004 | 0.0003 ± 0.0002 |
| miR-T4-3p-2 | 0.002 ± 0.001 | 7.97E-05 ± 0.0001 | ND | ND |
| miR-VP-3p-1 | 0.012 ± 0.016 | 0.002 ± 0.003 | ND | ND |
| miR-VP-3p-2 | 0.011 ± 0.012 | 0.002 ± 0.003 | 0.002 ± 0.003 | ND |

**a**

Supplementary Figure S1. EBOV miRNAs are detectable in longitudinally-collected NHP samples. EBOV-encoded miRNAs were detected in plasma samples from both (a) rhesus macaques exposed to aerosolized EBOV/Kikwit (n=3) and (b) cynomolgus macaques intramuscularly injected with EBOV/Makona (n=3). Data are presented as the mean ± SD. Viral loads were determined using an RT-qPCR assay targeted to the EBOV glycoprotein, and the miRNA was amplified with an LNA-containing primer set. Total RNA was extracted from 50 μL of NHP plasma and eluted into 50 μL (rhesus macaque samples) or 12 μL (cynomolgus macaque samples).

**b**

Supplementary Figure S2. EBOV miRNAs are present in whole blood from infected BALB/c mice. The ten candidate EBOV miRNAs were observed in mouse whole blood samples collected during daily terminal bleeds (mouse n=3 for each timepoint). Mice were injected intraperitoneally with 1000 PFU of mouse-adapted EBOV/Mayinga. Viral load was determined using an RT-qPCR assay which amplifies part of the viral glycoprotein sequence, and miRNAs were assayed with custom LNA-based primer sets. Total RNA was extracted from 175 μL mouse whole blood and eluted into 50 μL.

Supplementary Figure S3. EBOV miRNAs are detectable in acute-phase human EVD samples. Fifteen serum/plasma samples from human cases in the 2014 EBOV/Makona outbreak in Sierra Leone were assayed for EBOV-encoded miRNAs. Patient samples are arranged by day post-symptom onset (2-27 days).

**Supplementary Figure S4. LNA-based assays for EBOV miRNAs show good amplification characteristics.** Two custom LNA-based PCR primer sets (Exiqon, Inc.) were tested for each synthetic miRNA target. Assay linearity ranged from 0.9794 to 0.9998, and amplification efficiency (calculated from the slope of each standard curve) ranged from 83.83% to 122.51%. Synthetic miRNAs were serially diluted from 50 pM to 50 aM prior to cDNA synthesis, and PCR reactions were performed in triplicate at each concentration tested.

**Supplementary Figure S5. Comparison of Exiqon and TaqMan RT-qPCR assays for miR-VP-3p.** We used the same amounts of synthetic miR-VP-3p as input into each assay according to manufacturers’ instructions to construct the standard curves. The slope of the line for miR-VP-3p-1 is -3.494 (PCR efficiency=93.3%), for miR-VP-3p-2 is -3.414 (96.3%), and for the TaqMan assay is -3.63 (88.6%).

**References**

Chen, Zeliang, Hongwei Liang, Xi Chen, Yuehua Ke, Zhen Zhou, Mingjuan Yang, Ke Zen, Ruifu Yang, Chao Liu, and Chen-Yu Zhang. 2016. 'An Ebola virus-encoded microRNA-like fragment serves as a biomarker for early diagnosis of Ebola virus disease', *Cell Res*, 26: 380-83.

Herbert, Andrew S., Cristin Davidson, Ana I. Kuehne, Russell Bakken, Stephen Z. Braigen, Kathryn E. Gunn, Sean P. Whelan, Thijn R. Brummelkamp, Nancy A. Twenhafel, Kartik Chandran, Steven U. Walkley, and John M. Dye. 2015. 'Niemann-Pick C1 Is Essential for Ebolavirus Replication and Pathogenesis In Vivo', *mBio*, 6.

Liang, HongWei, Zhen Zhou, SuYang Zhang, Ke Zen, Xi Chen, and ChenYu Zhang. 2014. 'Identification of Ebola virus microRNAs and their putative pathological function', *Science China Life Sciences*, 57: 973-81.

Liu, Yuanwu, Jing Sun, Hongwen Zhang, Mingming Wang, George Fu Gao, and Xiangdong Li. 2016. 'Ebola virus encodes a miR-155 analog to regulate importin-α5 expression', *Cellular and Molecular Life Sciences*, 73: 3733-44.

Speranza, Emily, Louis A. Altamura, Kirsten Kulcsar, Sandra L. Bixler, Cynthia A. Rossi, Randal J. Schoepp, Elyse Nagle, William Aguilar, Christina E. Douglas, Korey L. Delp, Timothy D. Minogue, Gustavo Palacios, Arthur J. Goff, and John H. Connor. 2017. 'Comparison of Transcriptomic Platforms for Analysis of Whole Blood from Ebola-Infected Cynomolgus Macaques', *Scientific Reports*, 7: 14756.

Teng, Yue, Yuzhuo Wang, Xianglilan Zhang, Wenli Liu, Hang Fan, Hongwu Yao, Baihan Lin, Ping Zhu, Wenjun Yuan, Yigang Tong, and Wuchun Cao. 2015. 'Systematic Genome-wide Screening and Prediction of microRNAs in EBOV During the 2014 Ebolavirus Outbreak', *Scientific Reports*, 5: 9912.
